# Cadherin Expression, Vectorial Active Transport, and Metallothionein Isoform 3 Mediated EMT/MET Responses in Cultured Primary and Immortalized Human Proximal Tubule Cells

**DOI:** 10.1371/journal.pone.0120132

**Published:** 2015-03-24

**Authors:** Andrea Slusser, Chandra S. Bathula, Donald A. Sens, Seema Somji, Mary Ann Sens, Xu Dong Zhou, Scott H. Garrett

**Affiliations:** Department of Pathology, School of Medicine and Health Sciences, University of North Dakota, Grand Forks, ND, United States of America; UCL Institute of Child Health, UNITED KINGDOM

## Abstract

**Background:**

Cultures of human proximal tubule cells have been widely utilized to study the role of EMT in renal disease. The goal of this study was to define the role of growth media composition on classic EMT responses, define the expression of E- and N-cadherin, and define the functional epitope of MT-3 that mediates MET in HK-2 cells.

**Methods:**

Immunohistochemistry, microdissection, real-time PCR, western blotting, and ELISA were used to define the expression of E- and N-cadherin mRNA and protein in HK-2 and HPT cell cultures. Site-directed mutagenesis, stable transfection, measurement of transepithelial resistance and dome formation were used to define the unique amino acid sequence of MT-3 associated with MET in HK-2 cells.

**Results:**

It was shown that both E- and N-cadherin mRNA and protein are expressed in the human renal proximal tubule. It was shown, based on the pattern of cadherin expression, connexin expression, vectorial active transport, and transepithelial resistance, that the HK-2 cell line has already undergone many of the early features associated with EMT. It was shown that the unique, six amino acid, C-terminal sequence of MT-3 is required for MT-3 to induce MET in HK-2 cells.

**Conclusions:**

The results show that the HK-2 cell line can be an effective model to study later stages in the conversion of the renal epithelial cell to a mesenchymal cell. The HK-2 cell line, transfected with MT-3, may be an effective model to study the process of MET. The study implicates the unique C-terminal sequence of MT-3 in the conversion of HK-2 cells to display an enhanced epithelial phenotype.

## Introduction

The incidence of chronic kidney disease (CKD) is steadily rising and has reached epidemic proportions in the western and industrialized world. Clinicopathological studies have shown tubulo-interstitial fibrosis to be the hallmark of CKD progression [1–4]. This suggests that halting the progression of CKD disease could be achieved by stopping the progression or even by inducing remission of fibrosis. As recently reviewed by Prunotto and coworkers [5], renal fibrosis is defined as the scarring of the tubulo-interstitial space after kidney damage of any type, appears to be initiated at random in small areas that are preceded by interstitial inflammation, then expanding to become diffuse if drivers of fibrosis persist. Accumulation and proliferation of activated fibroblasts (myofibroblasts) in these small areas are linked to the risk of progression of fibrosis [6]. As reviewed, the exact source of renal myofibroblasts remains undefined and could include: migration of circulating fibrocytes to the site of the lesion, differentiation of local fibroblasts or pericytes, direct transformation of resident endothelial cells by the endothelial-mesenchymal transition (endoMT), or of resident epithelial cells through and epithelial-mesenchymal transition (EMT). Studies in experimental models have shown that it is the pericytes that respond to chronic injury and profibrotic signals through proliferation and differentiation into myofibroblasts [7, 8]. Fate tracing of pericytes has shown a direct contribution of these cells to renal fibrosis [9]. These studies, taken together, suggest a limited contribution for a direct conversion of renal epithelial cells, through the process of EMT, to produce the proliferative pool of fibroblast and myofibroblast cells seen during chronic kidney injury.

As highlighted in the review by Prunotto and coworkers [5], an indirect role for EMT in the progression of CKD can be proposed through alteration of the tubulo-interstitial microenvironment which can promote fibroblast proliferation and myofibroblast activation. This microenvironment would be produced by an alteration in epithelial to mesenchymal cellular cross talk produced by renal epithelial cells undergoing EMT upon renal injury. A role for an alteration in the microenvironment by renal cells undergoing EMT is consistent with early observations which showed that regions of active renal interstitial fibrosis exhibited a predominant peritubular as opposed to a perivascular distribution [10, 11]. In addition, some clinical features of CKD can be explained by a hypothesis that tubular epithelial cells can relay fibrogenic signals to contiguous fibroblasts in diseased kidneys [12, 13]. However, a role for EMT of renal epithelial cells producing a pro-fibrotic microenvironment remains a hypothesis supported by general observations, but not one supported by mechanism. One means to study the possible role of EMT in renal epithelial cells and its relationship to a microenvironment promoting fibrosis is the use of human renal epithelial cell cultures to model the mechanistic processes underlying the EMT. An examination of the literature suggests that the HK-2 cell line is the most common human renal epithelial cell line used to model human renal EMT and related renal disorders. The HK-2 cell line was isolated by immortalizing and cloning a cell line from a primary culture of proximal tubule epithelial cells transduced with a construct containing the HPV16 E6/E7 genes [14]. The HK-2 cell line proliferates in a serum-free growth medium comprised of keratinocyte serum free medium (KSFM) supplemented with epidermal growth factor and bovine pituitary extract. The HK-2 cell line is available from the American Type Culture Collection (ATCC) with instructions for growth in a KSFM medium supplemented with epidermal growth factor (EGF) and bovine pituitary extract (BPE). The HK-2 cells were shown to have an epithelial morphology and to retain many markers of proximal tubule cells such as alkaline phosphatase, gamma glutamyltranspeptidase, leucine aminopeptidase, acid phosphatase, cytokeratin, α_3_β_1_ integrin and fibronectin. Functional markers of proximal tubule differentiation also retained were: cAMP responsiveness to parathyroid hormone, but not antidiuretic hormone; Na^+^ dependent, phlorizin sensitive glucose transport; and, the ability to accumulate glycogen.

The present study is designed to address several questions that might impact on the studies that use the HK-2 cell line as a model for renal EMT. The first question is if the conditions used for the growth of the HK-2 cell line could impact on the results of studies examining the mechanism/s underlying renal EMT. This question arises since the growth media conditions recommended in the original publication, and those suggested by the ATCC, have not been employed in many investigations. A cursory review of the literature discloses that many studies that employ this cell line to address human renal EMT grow the HK-2 cells in various growth formulations containing fetal calf serum [15–24] as opposed to those using the recommended growth medium [25–29]. One could speculate that a growth media containing serum would in itself promote EMT and provide a more profibrotic growth environment that a growth media without serum. To examine this question, the present study determines the expression of two common markers of EMT, E-cadherin and N-cadherin, as a function of the growth media composition used for the growth of the HK-2 cells. In addition, previous studies from this laboratory demonstrated that the HK-2 cell line grown on serum-free growth medium had lost the ability for vectorial active transport as noted by a lack of dome formation and transepithelial resistance [30, 31]. The present study is also designed to determine if the loss of vectorial active transport was influenced by the presence or absence of serum in the growth medium of the HK-2 cells.

A second question explored in the present study is to begin to define the degree that the immortalization process used in the isolation of the HK-2 cells advanced the process of EMT compared to the parental cell line. This question is important since it is possible that the HK-2 cells may have already undergone a significant degree of the EMT. Evidence for this concept comes mainly from studies by this laboratory which developed the serum-free culture conditions for the growth of mortal cultures of human proximal tubule (HPT) cells that retain many of the differentiated features of the renal proximal tubule [32–34]. Similar cultures are now currently available from a number of commercial suppliers along with kits to support serum-free cell growth (some proprietary). In two related studies, this laboratory compared several properties of the HPT and HK-2 cells when grown on identical serum-free growth media [30, 31]. It was shown that when compared to the HPT cell line, the HK-2 cells had lost the capacity for vectorical active transport as noted by the inability to form doming structures [30]. In agreement with the absence of domes, the HK-2 cells, when compared to the HPT cells, were also shown to generate no transepithelial resistance and to lack the presence of tight junctions [30]. A corresponding analysis of E- and N-cadherin expression between the cell lines demonstrated a decrease in E-cadherin and an increase in N-cadherin when the HK-2 cells were compared to the HPT cells [31]. The determination of the E- and N-cadherin protein levels were qualitative in this past study and a goal of present study is to quantify the differences in E- and N-cadherin expression between the cell lines and to determine if additional differences exist in cell-to-cell junctional communication. The goal is to confirm that early features of EMT, such as the loss of cell-to-cell junctional integrity and a shift between E- and N-cadherin have already taken place in the HK-2 model system.

The above studies [30, 31] also demonstrated that the HK-2 cell line could undergo a reversal of the EMT process (MET) and regain vectorial epithelial transport, undergo a switch in E-, N, and K-cadherin expression, and regain junctional integrity upon stable transfection with the gene encoding the 3^rd^ isoform of the metallothionein protein (MT-3). A final goal in the present study is to define the functional domain of MT-3 eliciting increased MET in HK-2 cells.

## Materials and Methods

### Immunohistochemical Localization of E- and N-Cadherin Expression in Human Kidney

Tissue sections for the immunohistochemical analysis of E- and N-cadherin expression in human kidney were obtained from archival paraffin blocks of previously completed patient diagnostic procedures. These archival specimens contained no patient identifiers and use was approved by the University of North Dakota Internal Review Board. Three cases from renal cell carcinomas were utilized in the present analysis and areas of normal kidney were selected for use by a diagnostic pathologist. These archival specimens were routinely fixed in 10% neutral-buffered formalin for 16–18 h and were transferred to 70% ethanol and subsequently dehydrated in 100% ethanol. The dehydrated tissues were cleared in xylene, infiltrated, and embedded in paraffin. Serial sections of formalin fixed and paraffin embedded tissues were cut at 5 μm, deparaffinized in xylene and rehydrated in graded ethanol and water. Prior to immunostaining, antigen retrieval was performed by immersing the sections in Dako Target Retrieval Solution (Code S1699) and heated in a steamer for 20 minutes. The sections were allowed to cool at room temperature for 30 minutes and immersed in TBST (Tris-buffered saline with 0.1% Tween 20) for 10 minutes. The E and N-cadherin were detected by incubating the sections with anti-E cadherin (Santa Cruz; 1:100) and anti-N cadherin (Zymed; 1:100) for 30 minutes at room temperature. The identities of the tubular elements chosen for subsequent microdissection were identified by the differential immunostaining of sections for aquaporin-1 and calbindin [35–37]. Aquaporin-1 and calbindin were detected by incubating sections with anti-aquaporin (Ab9566, Abcam, Cambridge, MA, 1:400) and anti-calbindin (McAb 300, Swant, Switzerland, 1–800) for 30 minutes at room temperature. The sections were then incubated with Dako Envision+ Dual Link System (Code K4061) for 30 minutes at room temperature. Liquid diaminobenzidine (DAB) was used for visualization. After counterstaining with hematoxylin, the slides were rinsed in distilled water, dehydrated in graded ethanol, cleared in xylene, and coverslipped.

### Determination of E- and N-Cadherin mRNA Expression in Human Kidney Proximal Tubules Using Laser-Capture Microdissection

The parafin-embedded specimens were also used to determine the expression of E- and N-cadherin mRNA in proximal tubule cells of human kidney. Five μm thick sections were cut and mounted onto sterile plain glass slides. The slides were heated at 60°C for 25 min followed by incubation in xylene solution for 5 min and transferred into fresh xylene solution for an additional 5 min to deparaffinize the sections. The slides were then washed with 100% ethanol for 30 sec followed by sequential incubation for 30 sec in 100% ethanol, 95% ethanol, and 70% ethanol. The slides were then washed for 30 sec with distilled water and stained with hematoxylin for 30 sec. The slides were washed with distilled water followed by sequential washes with 70% ethanol and 95% ethanol for 60 sec each. The sections were incubated in eosin solution for 30 sec, washed with 95% ethanol for 60 sec, and then with 100% ethanol for 60 sec. The slides were incubated in xylene for 5 min to ensure the dehydration of the sections and excess solution was drained by touching the corner of the slide to a particle-free paper tissue. The slides were air-dried for 2 min to ensure the evaporation of xylene and were then used for laser capture microdissection. The PixCell II LCM system (Arcturus Engineering Inc., Mountain View, CA) was used and proximal tubules were identified by the diagnostic pathologist for microdissection. The microdissected samples were collected on the thermoplastic film of CapSure HS LCM caps (Arcturus Bioscience, Mountain View, CA). Total RNA was isolated following the protocol of RNAqueous—Micro Kit (Life Technologies, Carlsbad CA). The thermoplastic film containing the captured proximal tubule cells was incubated with 100 μl of lysis solution for 30 min at 42°C. Then 3 μl of LCM additive was added to the lysate. The lysate was briefly centrifuged followed by the addition of 1.25 volume of 100% ethanol. The lysate was passed through a pre-wetted microfilter cartridge by centrifugation at 10,000 x g for 1 min. The filter was washed with 180 μl of wash solution and the flow- through was discarded followed by two additional washes with 180 μl of wash solution. The flow-through was discarded and the filter was centrifuged for 1 min to remove the residual fluid. The filter was transferred into a new sterile tube and incubated for 5 min with 10 μl of elution solution preheated to 95°C. The RNA was eluted by centrifugation at 13,000 x g for 1 min and used in real-time reverse transcription polymerase chain reaction (RT-PCR).

The measurement of N-cadherin and E-cadherin was assessed using real time RT-PCR and commercially available primers (Qiagen Company, Valencia, CA) as described previously [31]. Real time PCR was performed utilizing the SYBR Green kit (Bio-Rad Laboratories) with 2 μl of cDNA, 0.2 μM primers in a total volume of 20 μl in an iCycler iQ Real-Time Detection System (Bio-Rad Laboratories). Amplification was monitored by SYBR Green fluorescence. Cycling parameters consisted of denaturation at 95^°^C for 15 seconds, annealing at 55^°^C for 30 seconds and extension at 72 ^°^C for 30 seconds which gave optimal amplification efficiency of each cadherin isoform. The relative levels of the three triplicates were then averaged for the final reported value (± SE) and reported as relative mRNA levels.

### Cell Culture

Stock cultures of HPT cells for use in experimental protocols were grown using serum-free conditions as previously described by this laboratory [32, 33]. The growth formulation consisted of a 1:1 mixture of Dulbecco's modified Eagles' medium (DME) and Ham's F-12 growth medium supplemented with selenium (5 ng/ml), insulin (5 μg/ml), transferrin (5 μg/ml), hydrocortisone (36 ng/ml), triiodothyronine (4 pg/ml) and epidermal growth factor (10 ng/ml). The cells were fed fresh growth medium every 3 days and were subcultured 1:2 at confluence (normally 3–6 days post subculture) using trypsin-EDTA (0.05% – 0.02%). HK-2 cells were obtained from the American Type Culture Collection, expanded following recommended culture conditions, and aliquots stored under liquid nitrogen. For studies directly comparing properties with HPT cells, the HK-2 cells were cultured under identical growth conditions using the serum-free growth media and subculture conditions described above for the HPT cells [30, 31]. To determine the effects of serum-free verses serum-contain growth medium on the morphology and expression patterns of the HK-2 cells, the HK-2 cells were grown to confluence on the serum-free medium as suggested by the original publication by Ryan and coworkers [14]. At confluence, the serum-free growth media of the HK-2 cells was replaced by the new media formulations and 24 hrs later the cells were subcultured using the new media formulations. The cells were allowed to grow under these new conditions for a minimum of 2 passages before use in experimental protocols.

### Transepithelial resistance

Measurement of transepithelial resistance was preformed as described previously [31]. Briefly, cells were seeded at a 2:1 ratio in triplicate onto 30-mm-diameter cellulose ester membrane inserts (Corning) placed in six-well trays. Beginning on the third day postseeding, transepithelial resistance (TER) was measured every other day with the EVOM Epithelial Voltohmmeter (World Precision Instruments, Sarasota, FL) with a STX2 electrode set according to the manufactures instructions. The resistance of the bare filter containing medium was subtracted from that obtained from filters containing cell monolayers. Two sets of four readings were taken at two different locations on each filter. The development of the monolayer in parallel six-well trays was monitored for dome formation. The experiment was repeated twice in triplicate and the final result reported as the mean ± SE.

### Real Time Analysis of E-Cadherin, N-Cadherin, and Connexin 32 mRNA Expression in Cultured Cells

The method used for the preparation of total RNA and the analysis of E- and N-cadherin has been described previously [31]. Primers for connexin 32 were obtained commercially (Qiagen Company, Valencia, CA).

### Western and ELISA Analysis of E- and N-Cadherin Expression

The determination of E- and N-cadherin by western blot has been described previously [31]. A sandwich ELISA method was used to quantify the expression of E- and N-cadherin by the cultured cell lines. Both ELISA materials were obtained from Abnova (E-cadnerin (#KA0433) and N-cadherin (Abnova, #KA1135). Plates were pre-coated with capture antibodies against either human E-or N-cadherin. Following initial titration to determine the appropriate net amount of protein to load per well, a range of 1–25 ug of total protein (100 μL/well) was used for analysis. The plate was covered and incubated at 37°C for 90 minutes (E-cadherin) or 2 hours (N-cadherin). The plate contents were discarded and incubated with 100 μL of biotinylated polyclonal antibody against either E-or N-cadherin diluted (1:100) in antibody dilution buffer for 1 hour at 37°C. The contents of the plate were discarded and each well was washed three times with 10 mM TBS. The plate was incubated with pre-warmed avidin-biotin-peroxidase complex (ABC) prepared 1:100 with ABC-dilution buffer at 37°C for 30 minutes. The contents were discarded and the plate was washed 5 times. The plate was then incubated with 90 μL of pre-warmed 3, 3′, 5, 5′ tetramethylbenzidine (TMB) substrate solution for 15 minutes at 37°C. Finally, the reaction was quenched by the addition of acidic TMB stop solution halting the colorimetric development. The plate was then analyzed by reading the absorbance of each well on a Biotek plate reader at 450 nm. The data was analyzed via MasterPlex software using 5 parameter logistics to determine the amount of E- or N-cadherin present in each well.

### Western Analysis of Connexin 32

Western analysis was performed as before [31], with a connexin 32 specific antibody (Life Technologies, clone CX-2C2) at 0.5 μg/ml.

### Protein isolation from cells grown on Transwell inserts

Confluent cultures were harvested 7 days after reaching confluency. The inserts were washed three times with ice cold PBS prior to the addition of cold complete RIPA lysis buffer (Santa Cruz Biotechnology, Dallas, TX). The apical chamber was gently scraped to collect the cell lysate and rocked for 30 minutes at 4°C. The lysate was centrifuged 13,000*g* at 4°C for 15 minutes to pellet cellular debris. The supernatant was transferred to a fresh, cold microfuge tube and stored at −80°C until quantification and western blot analysis. Parallel cultures were grown on tissue cultured treated 6-well plates and treated identically to the inserts.

### Stable Transfection

**Fig 1:**
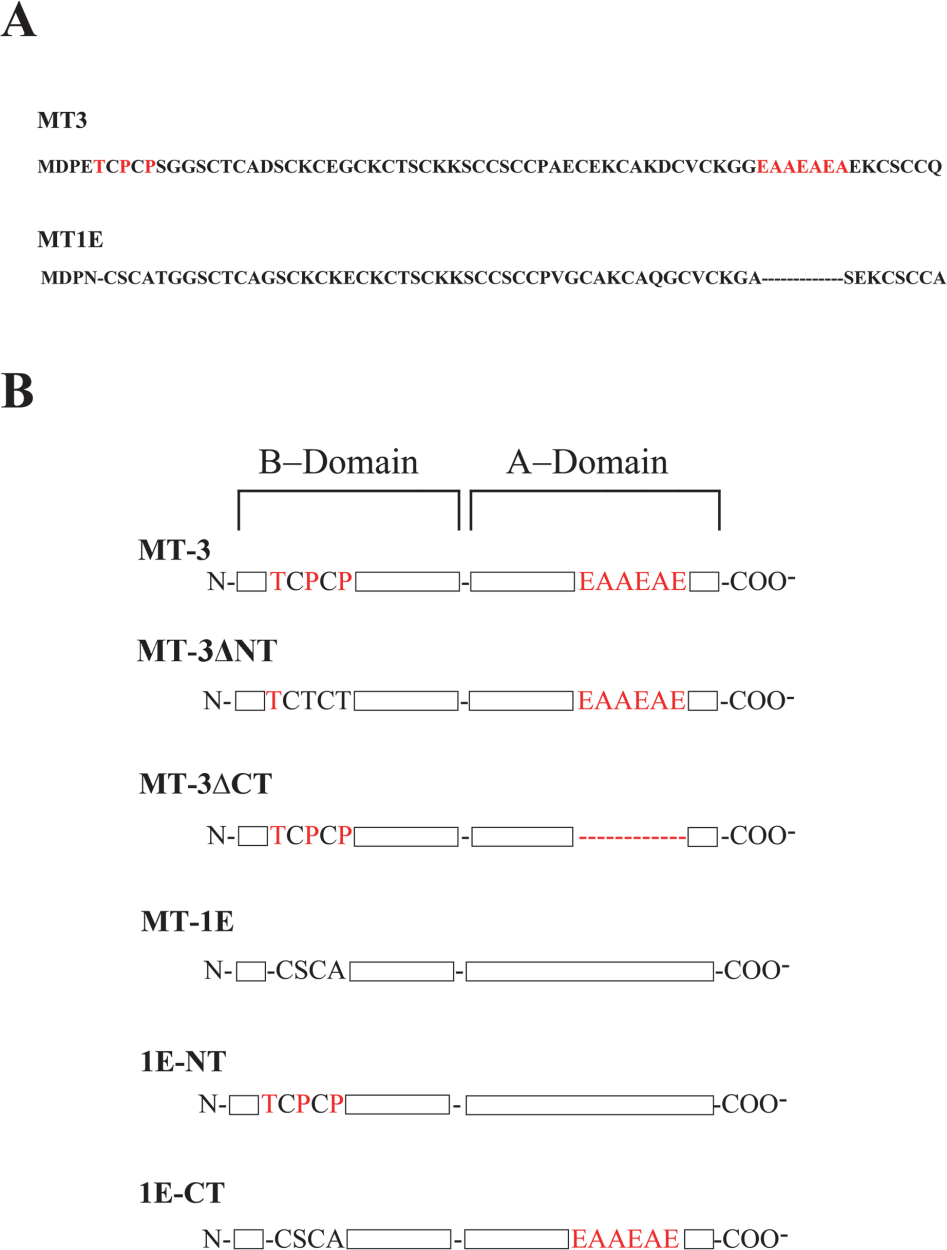
Mutated MT constructs. A) Amino acid sequence of human MT-3 compared with human MT-1E. Shown in red are the MT-3 specific sequences that are not exhibited in any mammalian MT 1/2 isoform. The N-terminal TCPCP sequence of MT-3 has been shown to confer the unique biological activity of neuronal growth inhibitory activity [38]. These two sequences were independently deleted from MT-3 and inserted into the MT-1E isoform to test for the conference of vectorial active transport. B) Schematic diagram of the various metallothionein constructs with: MT-3 denoting the wild-type sequence; MT-3-ΔNT, the two essential prolines were converted to threonines; MT-3ΔCT, the EAAEAE C-terminal sequence was deleted from MT-3; MT-1E, wild-type 1E isoform of metallothionein; 1E-NT, the N-terminal sequence of MT-3 was inserted into the corresponding position of MT-1E; 1E-CT, the MT-3 C-terminal sequence EAAEAE was inserted into the corresponding position of MT-1E.

The HK-2 cells were transfected with wild type MT-3, MT-3 where the unique N-terminal region was altered (ΔNT), and MT-3 where the unique C-terminal sequence was removed from MT-3 (ΔCT) (Fig. 1). The HK-2 cells were also transfected with MT-1E, MT-1E where the unique C-terminal sequence of MT-3 was added to the corresponding region of MT-1E (1E-CT), and MT-1E in which the sequence of the N-terminal domain was changed to the MT-3 sequence consensus (1E-NT) (Fig. 1). The Gateway cloning system (Invitrogen, Carlsbad, CA) was used for the production MT-3 site-directed mutants. The MT-3 and MT-E coding sequences were cloned into the pENTR vector and site-directed mutagenesis was performed by GeneScript (Piscataway, NJ). The EAEAAE sequence was deleted from the C-terminal domain of MT-3 and this same sequence was inserted in the analogous position of MT-1E, producing the MT-3ΔCT and 1E-CT constructs respectively. The N-terminal sequence of MT-1E (MDPNCSCA) was converted to an MT-3-like sequence (MDPNTCPCP) for the 1E-NT construct. For the mutagenesis of this N-terminal sequence, the last two prolines in the above sequence were converted to threonine for the MT-3-NT mutant. The threonine insert and prolines 7 and 9 of MT-3 were found to be necessary and sufficient to confer growth inhibitory activity and the substitution of the prolines to threonine is sufficient to eliminate this activity [38]. Each metallothionein sequence within the pENTER vector was moved to the pcDNA6.2/V5-DEST destination vector using the Gateway LR recombination reaction. For those constructs that lack the EAEAAE sequence, the epitope of the MT-3 antibody, the stop codon was removed allowing for the translation of the V5 tag on the C-terminus for alternative protein detection. Expression of each clone was assessed using immunodot blot as reported previously (Kim et al. 2002).

The HK-2 cells were transfected with the E-cadherin coding sequence using the E-cadherin ORF cloned in pENTR 221 vector and was obtained from Invitrogen (Invitrogen, Carlsbad, CA). The E-cadherin gene was transferred into pcDNA 6.2/V5-DEST vector by LR recombination reaction (Invitrogen, Carlsbad,CA). The DNA constructs were linearized with *BspH* I and E-CDH ORF was linearized with *Bcg* I (New England BioLabs, Ipswich, MA) before transfection. The HK-2 cells were transfected using the Effectene transfection reagent (Qiagen, Valencia, CA) following the manufacturer’s protocol at a ratio of 1:10 plasmid to Effectene ratio, and the lipid complexes were added to the cells at 2 μg of DNA per 9.6 cm^2^ well of a 6-well culture plate. For antibiotic selection, transfected cells were seeded 1:10 and clones were selected in 3 μg/ml blasticidin and clones were isolated with cloning rings and propagated in culture medium containing 3 μg/ml blasticidin.

### Statistical Analysis

All experiments were performed in triplicate and repeated at least three times (unless otherwise stated) and the results are expressed as the standard error of the mean. Statistical analyses were performed using GraphPad Prism 5 software (La Jolla, CA, USA) using separate variance t-tests, ANOVA with Tukey post-hoc testing. Unless otherwise stated, the level of significance was 0.05.

## Results

### Expression of E- and N-Cadherin in Human Kidney Proximal Tubules

**Fig 2:**
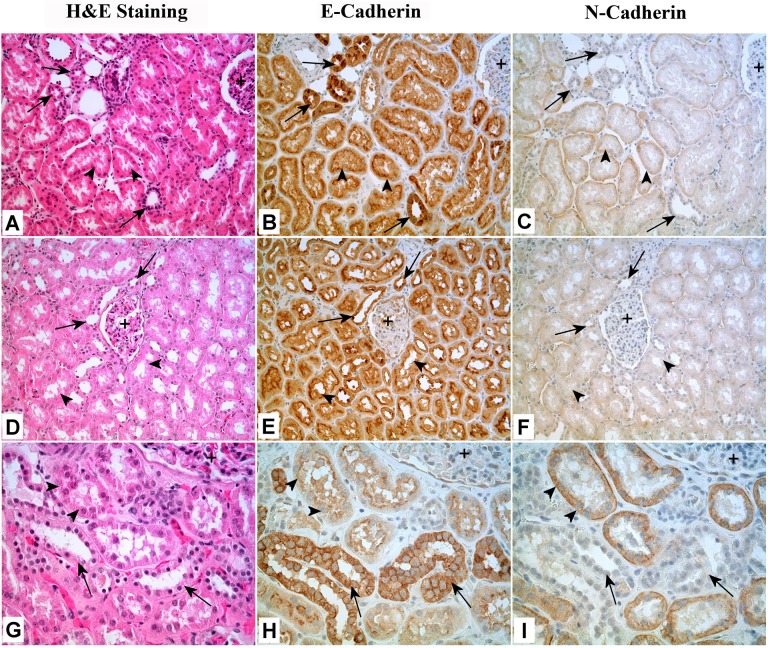
Immunohistochemical staining of E- and N-Cadherin in human kidney. A, B, C: The majority of the tubules in the image are proximal tubules (A, arrow heads). These tubules are moderately positive for E-cadherin with stronger staining on the luminal border (B, arrow heads). Proximal tubules are also moderately positive for N-cadherin (C, arrowheads). A few of distal tubules are strongly positive for E-cadherin (B, arrows), but negative for N-cadherin (C, arrows). On the upper right corner is a part of glomerulus (A, +), which shows very weak staining of E-cadherin and no staining of N-cadherin (B, C, +). 200X. D, E, F: In the center of the image is a glomerulus (D, +), which is very weakly positive for E-cadherin (E, +) but negative for N-cadherin (F, +). Almost all of the tubules around the glomerulus are proximal tubules, which are moderately to strongly positive for E-cadherin, especially on the luminal or apical border (E, arrowhead), but only weakly positive for N-cadherin (F, arrowhead). The arrows indicate the only 2 or 3 distal tubules, which are strongly positive for E-cadherin (E, arrows), but negative for N-cadherin (F, arrows). 200X. G, H, I: At higher magnification, it can be clearly seen that the E-cadherin is present around the whole epithelial cells of distal tubules (H, arrows) with stronger staining in the cell borders (H, arrows); while in proximal tubules, E-cadherin is mainly located in the luminal borders (H, arrowheads). For N-cadherin, it is only present in proximal tubules, and its signal is mainly located in the basolateral sides of the tubules (I, arrowheads). Distal tubules are totally negative for N-cadherin (I, arrows). A small part of glomerulus can be seen in upper right corner (G, H, I, +). X400

**Fig 3:**
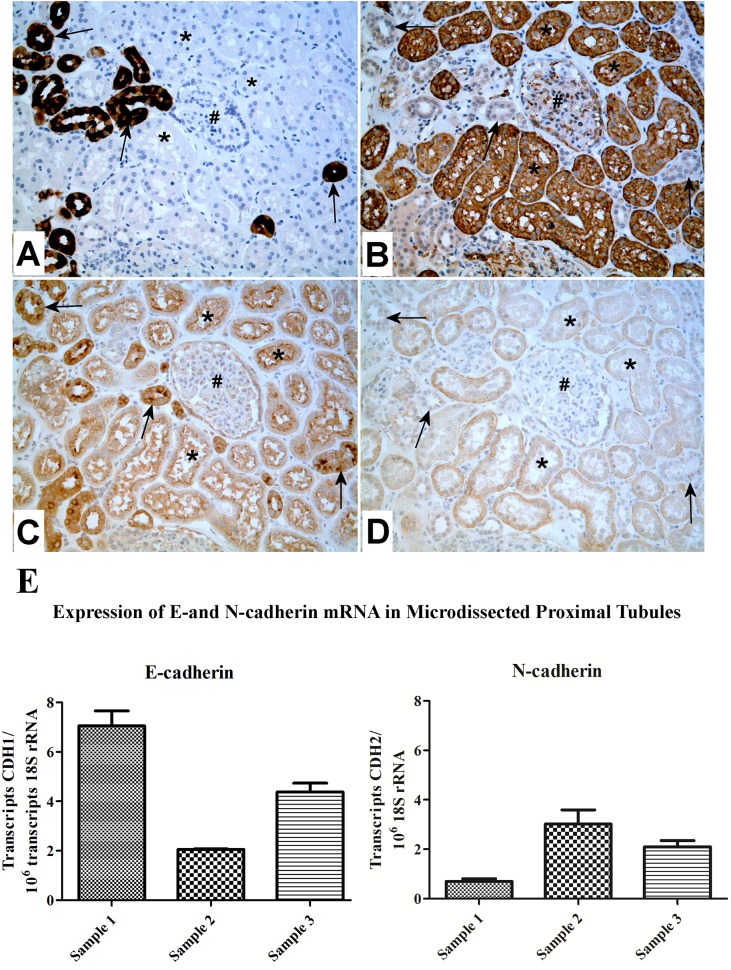
Expression of E- and N-cadherin mRNA in microdissected proximal tubules. A and B: Identification of proximal tubules by Immunohistochemical staining in matched area of normal kidney for calbindin (A)(distal) and aquaporin-1 (B) (proximal). C and D: Verification of E-cadherin (C), N-cadherin (D) expression. The proximal tubules (marked by *) are moderately to strongly positive for E-cadherin (mainly in the luminal side), moderately positive for N-cadherin (mainly in basolateral side), negative for calbindin, and diffusely strongly positive for aquaporin-1. In contrast, the distal tubules (pointed by arrows) show diffuse, strong staining of E-cadherin and calbindin, but no staining or faint staining of N-cadherin and aquaporin-1. The glomeruli (indicated by #) are weakly stained by E-cadherin and aquaporin-1, but negative for N-cadherin and calbindin. Original magnification: X200. Only one example of the serial sections is presented, and all three specimens gave identical results. E: Expression of E- and N-cadherin mRNA in microdissected proximal tubules. RNA was purified from laser-capture microdissected proximal tubules identified in sections of formalin-fix, paraffin-embedded tissue obtained from human renal cortex. Levels of RNA for E- and N-cadherin were determined quantitatively using real-time PCR and normalized to the levels of 18S rRNA also assessed with real-time PCR. The levels of each cadherin isoform were determined in triplicate and expressed as the number of detected cadherin transcripts per million transcripts of rRNA. The results are expressed as the mean of triplicate determinations (± SE).

To confirm the expression of E-cadherin in the proximal tubule, serial sections were prepared from three independent specimens of human kidney in order to compare routine H&E histology with expression of E- and N-cadherin. In proximal tubules, staining for E-cadherin was moderate in intensity for all three specimens with staining concentrated on the luminal or apical borders of the cells (Fig. 2 A, B; D, E; G, H). In distal tubules, staining for E-cadherin was usually stronger than that observed for proximal tubules and staining was present around the entire epithelial cell (Fig. 2 A, C; D, F; G, I). In proximal tubules, staining for N-cadherin was also moderate in intensity and often localized to the basolateral side of the tubules (Fig. 2 A, C; D, F; G, I). Distal tubules were negative for the expression of N-cadherin (Fig. 2 A, C; D, F; G, I). The glomeruli were negative for the expression of N-cadherin and negative to very weakly positive for the expression of E-cadherin. Microdissection was utilized to determine the expression of E- and N-cadherin mRNA in proximal tubules isolated from tissue sections obtained from the three archival specimens of human kidney. Serial sections were stained for calbindin and aquaporin to identify that proximal tubules were selectively chosen for microdissection (Fig. 3A—B). Immunohistochemisstry for E- and N-cadherin verified the proximal and distal tubule distribution seen in Fig. 2 (Fig. 3 C, D). The results of this determination showed that mRNA for both E- and N-cadherin was expressed in proximal tubules isolated from all three independent specimens (Fig. 3E). The levels of expression of E-cadherin varied between 2 and 7 transcripts and that of N-cadherin between 0.5 and 3 transcripts.

### Expression of E- and N-Cadherin in HK-2 Cells as a Function of Growth Medium Composition

**Fig 4:**
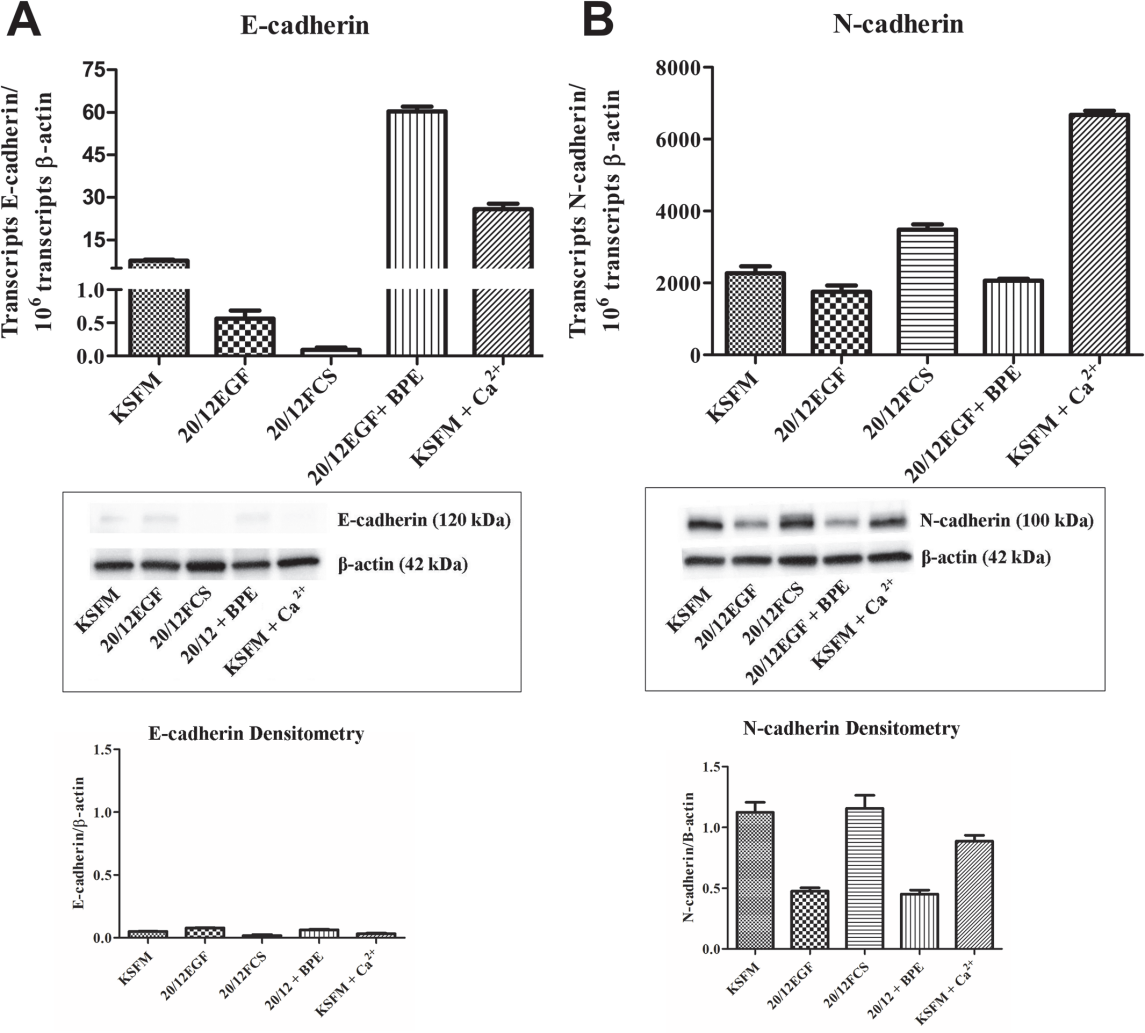
Expression of E- and N-cadherin in HK-2 cells as a function of growth medium. A) E-cadherin mRNA expression in HK-2 cells grown in various growth media. Expression was assessed with real-time RT-PCR and expressed as the number of transcripts per million transcripts of β-actin. Western analysis of E-cadherin is shown below the graph. See Materials and Methods for media formulations. B) N-cadherin mRNA expression in HK-2 cells grown in various growth media. Messenger RNA expression was assessed by real-time PCR. Western analysis of N-cadherin is shown below the graph.

In this analysis, the expression of E- and N-cadherin was determined on the HK-2 cell line when grown of three different growth media formulations. The first formulation was that developed by Ryan and coworkers [14], and suggested for use by the ATCC, that is composed of keratinocyte serum free media supplemented with 0.05 mg/ml BPE and 5.0 ng/ml EGF (designated KSFM). The second formulation tested was that used by Detrisac and coworkers [32] for the growth of human proximal tubule (HPT) cells and is composed of a a 1:1 mixture of DME and F-12 containing selenium (5 ng/ml), insulin (5 μg/ml), transferrin (5 μg/ml), hydrocortisone (36 ng/ml), triiodothyronine (4 pg/ml) and epidermal growth factor (10 ng/ml) (designated 20/12EGF). The third formulation was chosen to be representative of one of the various growth mediums used that contained fetal calf serum [20] and was a 1:1 mixture of DME and Ham's F-12 growth medium containing 10% fetal calf serum (designated 20/12FCS). In addition, it was also determined if adding 0.05 mg/ml of BPE to the 20/12EGF formulation and if raising the calcium concentration of the KSFM to that of 20/12 had any effect on the expression of E- and N-cadherin. The results of this determination showed that the expression of E-cadherin mRNA was very low in the HK-2 cells regardless of the growth media formulation, being on the order of 1 mRNA transcript per cell (Fig. 4A). The corresponding matching western blots showed only very faint bands corresponding to the expression of the E-cadherin protein (Fig. 4A). In contrast, the expression of N-cadherin mRNA was much higher in the HK-2 cells when compared to that of E-cadherin, with most conditions exhibiting hundreds of fold greater level of expression regardless of growth formulation (Fig. 4B). The N-cadherin protein was also prominent on western blots (Fig. 4B). Similar to that found for E-cadherin, the growth media composition had little effect on N-cadherin expression. Overall, the analysis demonstrated that the HK-2 cells have a low expression of E- compared to N-cadherin and that growth media composition has only a marginal influence on the levels of mRNA or protein expression.

### Comparison of E- and N-Cadherin Expression in HK-2 and HPT Cell Cultures

**Fig 5:**
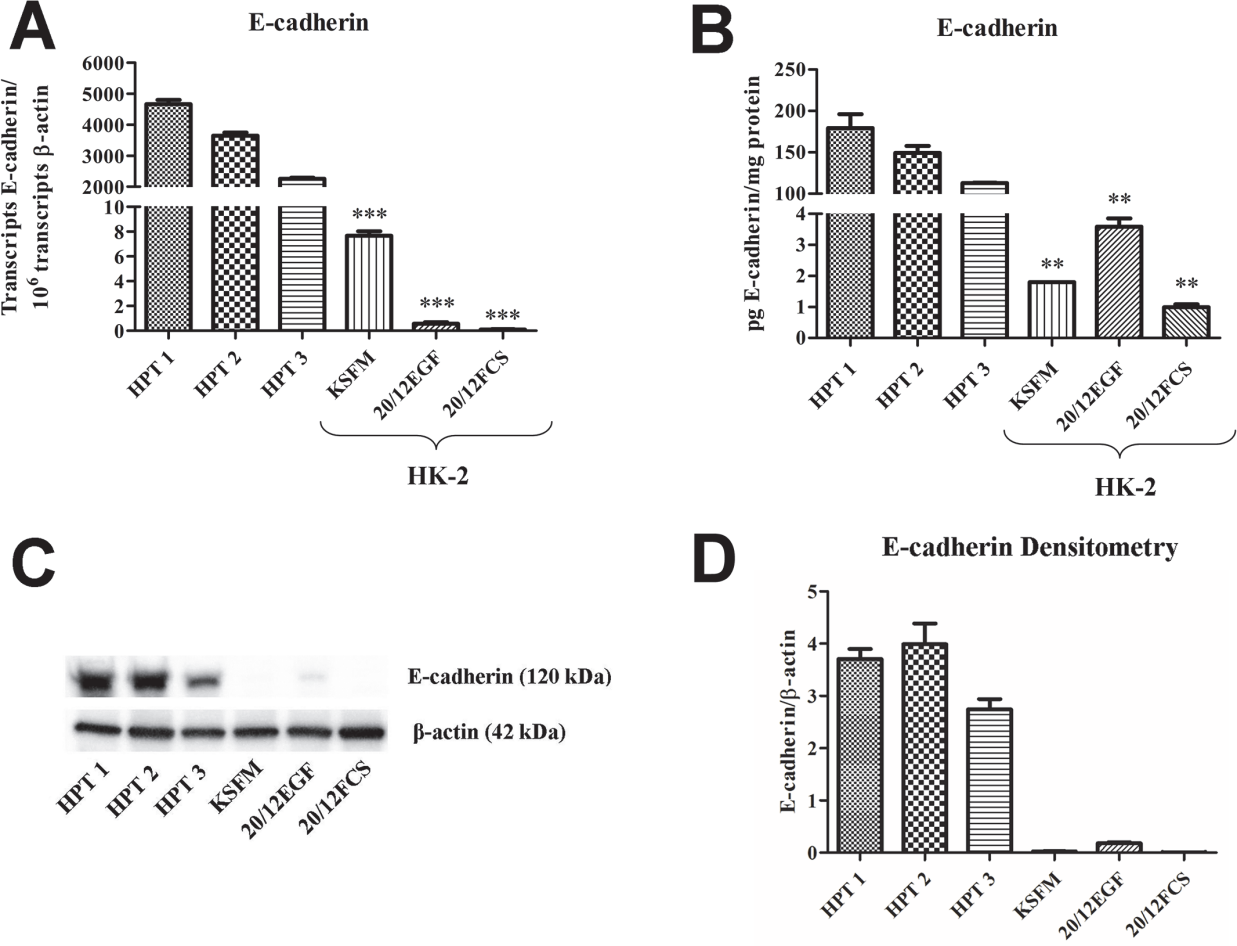
Comparison of E-cadherin expression in HPT and HK-2 cell cultures. A) Expression of E-cadherin mRNA in three independent HPT isolates (human proximal tubule cells) compared to that in HK-2 cells growth in three different media formulation. HPT cells were grown in the 20/12EGF formulation. B) Levels of E-cadherin protein measured quantitatively using an ELISA. C) Western analysis of E-cadherin in the identical cultures as in A and B. D) Graphical representation of the integrated optical densities of the western shown in C. Significant differences between HK-2 and each HPT isolate are designated ***p < 0.0001, of ** p< 0.001 as determined by one-way ANOVA with Tukey’s post-hoc test

**Fig 6:**
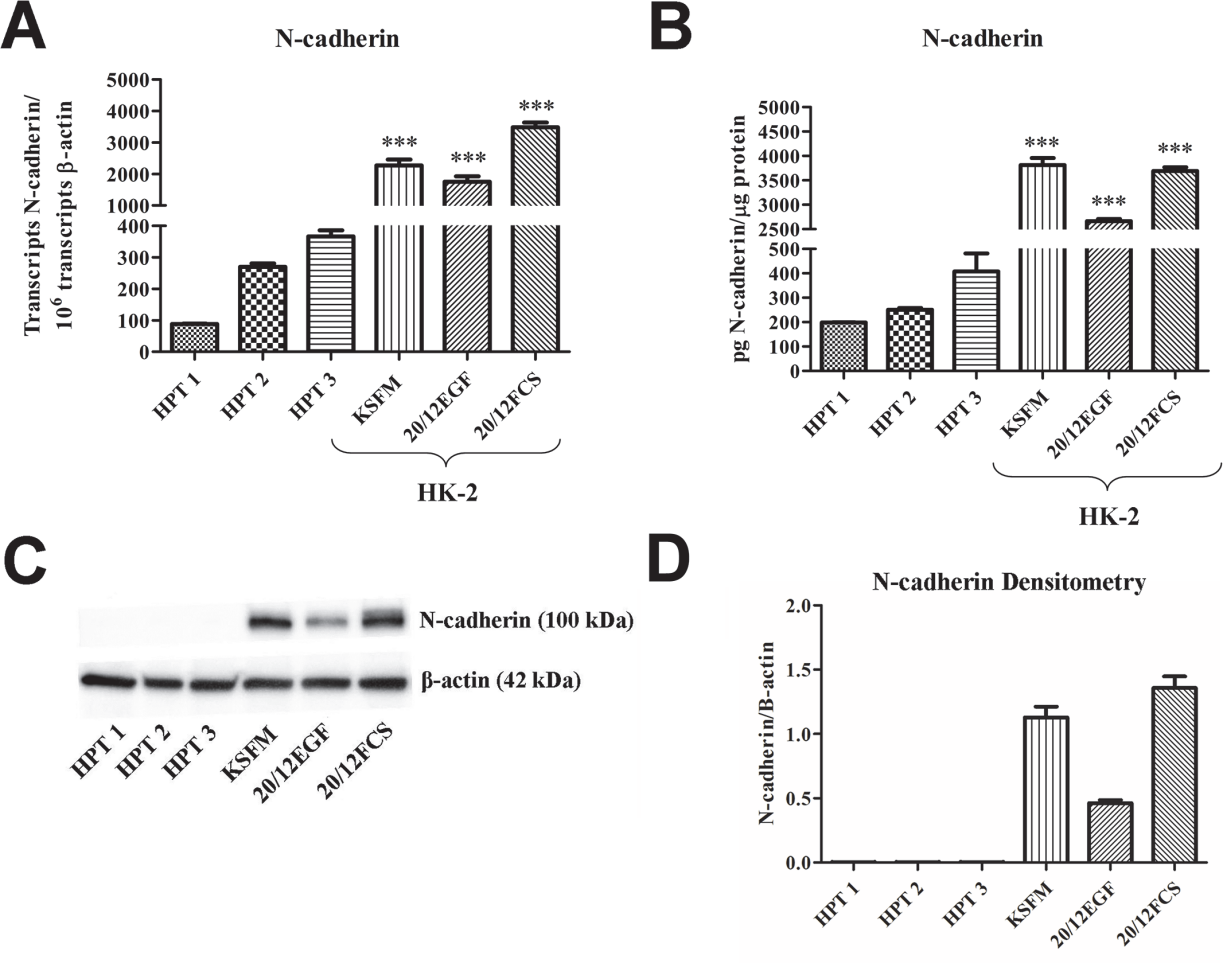
Comparison of N-cadherin expression in HPT and HK-2 cell cultures. A) Expression of N-cadherin mRNA in three independent HPT isolates (human proximal tubule cells) compared to that in HK-2 cells growth in three different media formulation. HPT cells were grown in the 20/12EGF formulation. B) Levels of N-cadherin protein measured quantitatively using an ELISA. C) Western analysis of N-cadherin in the identical cultures as in A and B. D) Graphical representation of the integrated optical densities of the western shown in C. Significant differences between HK-2 and each HPT isolate are designated ***p < 0.0001 as determined by one-way ANOVA with Tukey’s post-hoc test.

The expression of E- and N-cadherin was compared between the HK-2 and HPT cells at the level of mRNA and protein expression. Since the results of western analysis can be influenced by the development time used to produce the blot, an ELISA-based method was employed to quantify the amount of E- and N-cadherin that was present in both the HK-2 and HPT cells. Three independent isolates of HPT cells were used in the experiments and these were grown on 20/12EGF. The HK-2 cells were grown on KSFM, 20/12FCS, and 20/12EGF. The results of this analysis confirmed that the HPT cells produced E-cadherin mRNA in substantially higher amounts than the HK-2 cells regardless of growth media composition (Fig. 5A). The levels of E-cadherin mRNA in HPT cells was over 50 fold higher than levels in HK-2 cells. This difference in mRNA expression did translate to the amount of E-cadherin protein, with ELISA analysis showing a similar large (>50 fold) increase in E-cadherin protein in the HPT cells compared to the HK-2 cell line (Fig. 5B). This difference in protein level was also exemplified on a standard western as shown in Fig. 5C & D. An identical analysis of N-cadherin expression confirmed that N-cadherin mRNA expression was higher in the HK-2 cell line compared to the HPT cells (Fig. 6A). An analysis of N-cadherin protein expression also demonstrated that this difference did translate to the differences in the N-cadherin protein (Fig. 6B, C and D). However, the magnitude of the difference between N-cadherin protein expression between the HPT and HK-2 cell lines was much less (4 to 10 fold) than that found for the E-cadherin protein (Fig. 5B versus 6B), albeit, the smaller difference did not manifest on the western in Fig. 6C and D most probably due to the higher sensitivity of the ELISA in detecting low levels of N-cadherin in the HPT samples. Overall, the results demonstrate that the level of E-cadherin mRNA and protein is significantly higher in HPT cells compared to HK-2 cells and that the expression pattern is reversed for the expression of N-cadherin between the cell lines.

**Fig 7:**
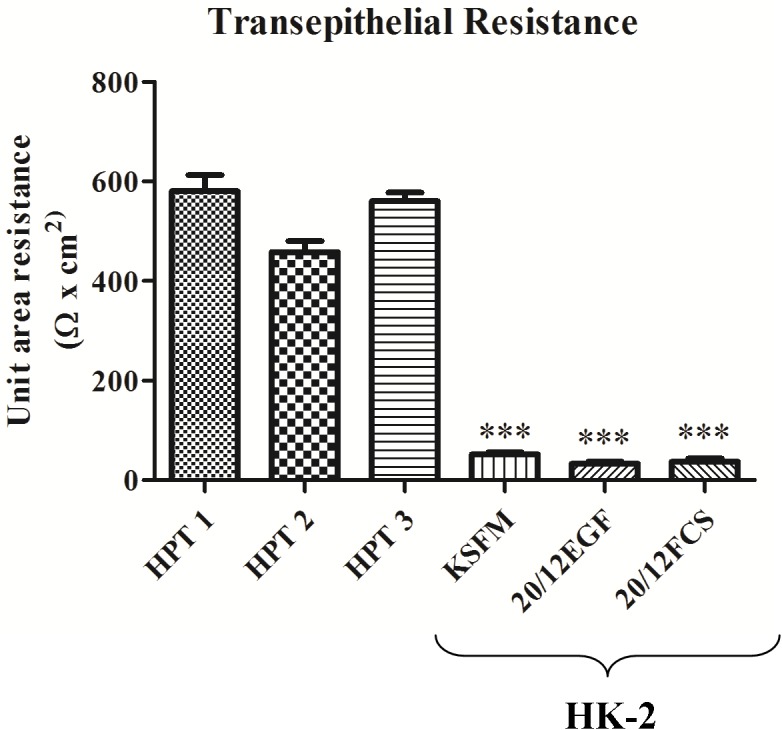
Comparison of Transepithelial resistance in HPT and HK-2 cells. Cells were grown on filter supports and the electrical resistance across the monolayer was measured. Resistance is expressed as Ohms-cm^2^. Three independent HPT isolates (human proximal tubule cells) are compared to that in HK-2 cells growth in three different media formulation. HPT cells were grown in the 20/12EGF formulation. Significant differences between HK-2 and each HPT isolate are designated *p < 0.0001 as determined by one-way ANOVA with Tukey’s post-hoc test.

**Fig 8:**
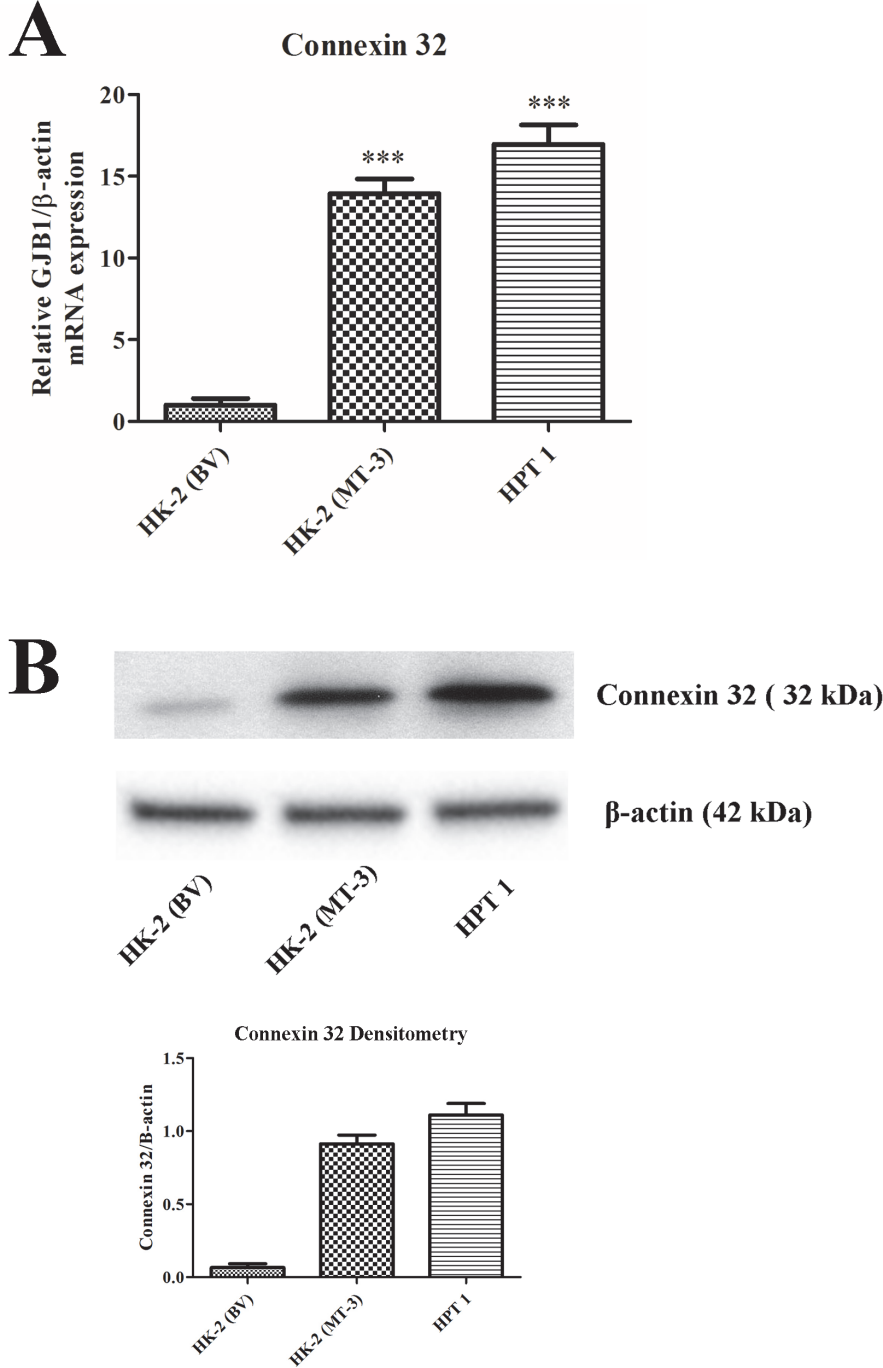
Connexin 32 expression in HK-2, HPT, and HK-2 cells expressing MT3. Messenger RNA of connexin 32 was assessed with real-time PCR and expressed as a fold increase of the HK-2 cells stably transfected with the blank vector. The change in connexin 32 expression was normalized to the change in β-actin expression. Western analysis of connexin 32 is shown below the graph.

The transepithelial resistance of the HK-2 and HPT cell monolayers was also determined to confirm that the growth media composition had no effect on the tight junctions between the cells. It was shown that the HK-2 developed no transepithelial resistance (TER), whereas, the HPT cell line developed a TER consistent with a “leaky” epithelium (Fig. 7). As a further validation for an alteration in cell-to-cell junctions, the HK-2(MT-3), HK-2(blank vector) and HPT cells were assessed for the expression of connexin 32. The results of this determination showed that HK-2(BV) had marginal expression of connexin 32 mRNA and protein, while the HK-2(MT-3) and HPT cells each showed significant expression of both connexin 32 mRNA and protein (Fig. 8A). This finds further validate the findings of altered cell-to-cell junctional responsibilities between the HK-2 and the HPT cell line and extend those observations to communication between adjacent cells. To validate that the patterns of E- and N-cadherin expression observed were not an artifact of growth on tissue culture treated plastic, HK-2, HK-2 (MT-3), and two isolates of HPT cells were grown on 30 mm Transwell inserts. Protein lysates were harvested from the cells on the Transwell inserts and parallel cultures grown on 6-well tissue culture treated plastic and subjected to western analysis for both E- and N-cadherin protein expression. The results of this experiment show that the expression level of theses cadherins was not altered from culturing in the Transwell-insert environment (S1 Fig.).

### Association of the Unique C-Terminal Domain of MT-3 With MT-3 Induced MET in HK-2 Cells

The goal of this experiment was to determine the functional domain of the MT-3 protein required for the MT-3 protein to re-establish vectorial active transport, produce an enhanced epithelial morphology, elicit a shift in the expression of E- and N-cadherin, and to increase the expression of connexin 32. Four constructs were used to transfect the HK-2 cells in this analysis (Fig. 1) and vectorial active transport judged by the appearance of domes and the generation of a transepithelial resistance. The first construct was MT-3 where the N-terminal sequence was been mutated, the second was MT-3 where the C-terminal sequence was mutated, the third was the MT-1E isoform where the unique C-terminal sequence of MT-3 was inserted into the sequence, and four the MT-1E isoform where the unique N-terminal sequence of MT-3 was inserted into the sequence. The HK-2 cell line carrying the blank vector or transfected with the MT-1E isoform served as controls.

**Fig 9:**
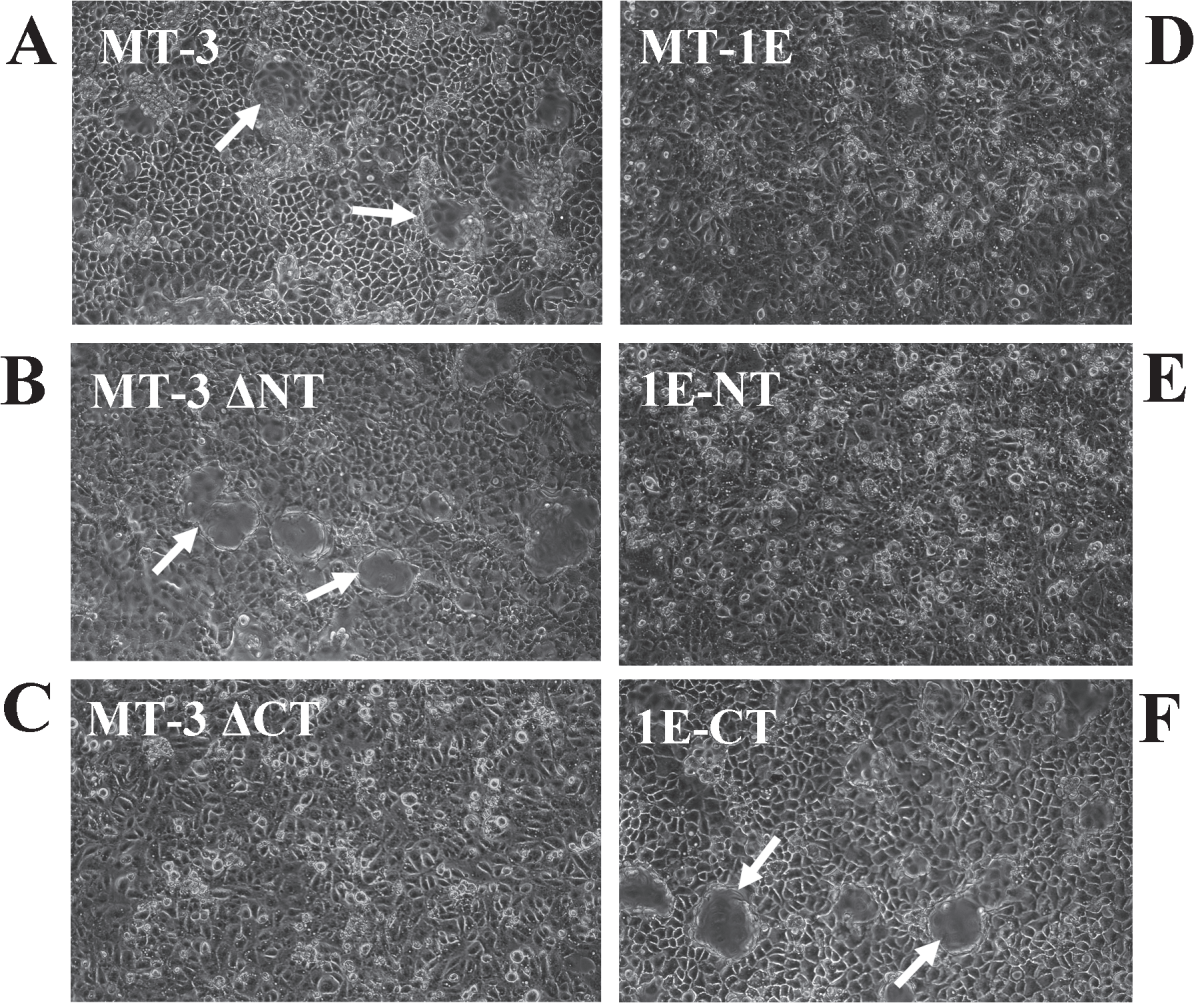
Effect of altered domains of metallothionein-3 on the formation of domes in HK-2 cells. To assess the domain of MT-3 that is responsible for the ability to confer dome formation, site-directed mutants of MT-3 were produced and the two unique domains of MT-3 were inserted into the non-doming MT isoform, MT-1E, as shown in Fig. 1. Each expression construct was stably transfected into HK-2 cells, and expressing clones were assessed for the ability to form domes. A) wild-type MT-3 showing dome formation when stably transfected, B) MT-3-ΔNT where the prolines in the N-terminal domain that confers growth inhibitory activity were converted to threonines, show that the ability to form domes was not compromised, C) MT-3ΔCT where the C-terminal EAAEAE sequence unique to the third isoform of metallothionein was deleted, shows the lack of dome formation, D) MT-1E, wild-type human metallothionein 1E, commonly expressed at high levels in many cell types exhibits no domes when stably transfected, E) 1E-NT, the N-terminal unique sequence of MT-3 was inserted into the corresponding position of MT-3 shows no conference of dome formation, and F) 1E-CT, the EAAEAE sequence of MT-3 was inserted in the corresponding position of MT-1E and when stably transfected into HK-2 cells confers dome formation.

**Fig 10:**
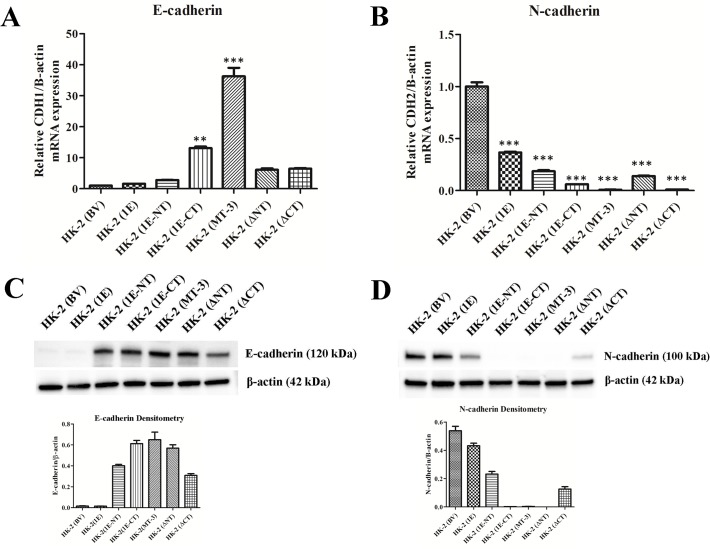
Effect of altered domains of metallothionein-3 on the expression of E- and N-cadherin in HK-2 cells. Messenger RNA of E-cadherin (A) and N-cadherin (B) were assessed with real-time PCR and expressed as fold change in expression versus HK-2 cells transfected with the blank vector. The change in E- or N-cadherin expression was normalized to the change in β-actin expression. Significant differences from HK-2 (BV) are designated as ** p < 0.001, *** p < 0.0001. Western analysis of E-cadherin (C) and N-cadherin (D) was conducted in identical cultures as in A and B. β-actin was used as a loading control and for densitometric normalization. Densitometry is shown below the corresponding blot.

**Fig 11:**
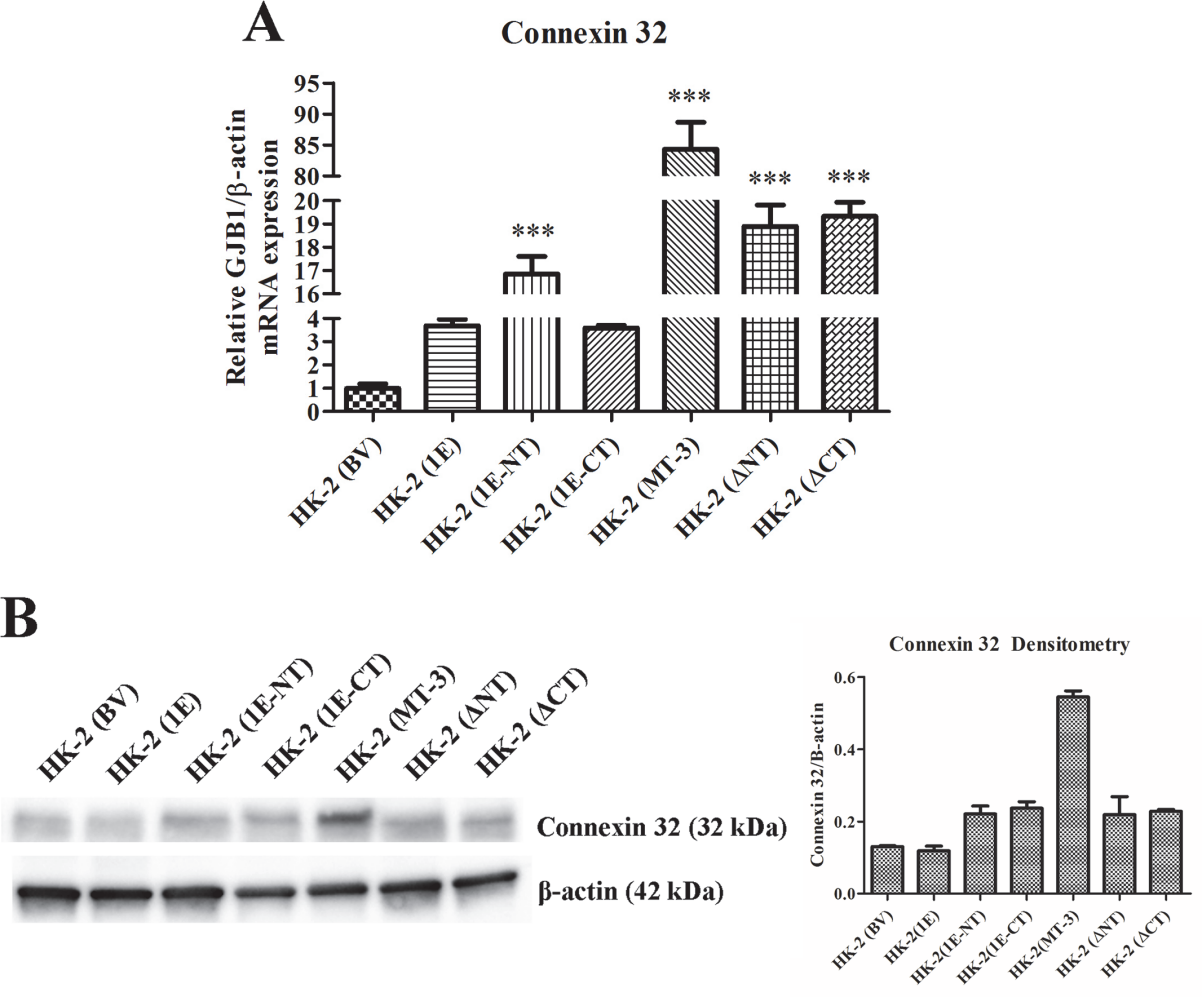
Effect of altered domains of metallothionein-3 on the expression of connexin 32 in HK-2 cells. A) Messenger RNA of connexin 32 assessed with real-time PCR and expressed as fold change in expression versus HK-2 cells transfected with the blank vector. The change in connexin 32 expression was normalized to the change in β-actin expression. Significant differences from HK-2 (BV) are designated as *** p < 0.0001. B) Western analysis of connexin 32 was conducted in identical cultures as in (A). β-actin was used as a loading control and for densitometric normalization. Densitometry is shown beside the corresponding blot.

The results of this determination demonstrated that the C-terminal sequence of MT-3 is required for the establishment of vectorial active transport when MT-3 is transfected and expressed in the HK-2 cell line. First, the mutation of the N-terminal sequence of MT-3 had no effect on the ability of HK-2 cells to form domes or generate a transepithelial resistance (Fig. 9 A,B; Table 1). In contrast, mutation of the C-terminal sequence of MT-3 abolished both dome formation and the transepithelial resistance of the monolayer (Fig. 9A,C; Table 1). The MT-1E isoform of MT does not contain either of the unique C-terminal or N-terminal sequences of MT-3 and transfection of HK-2 cells with MT-1E do not form domes in culture or develop a transepithelial resistance (Fig. 9D). The insertion of the N-terminal sequence of MT-3 into the MT-1E gene and subsequent transfection into the HK-2 cells does not result in dome formation or the generation of a transepithelial resistance (Fig. 9E, Table 1). In contrast, the insertion of the C-terminal sequence of MT-3 into the MT-1E gene and subsequent transfection into the HK-2 cells results in both dome formation and the generation of a transepithelial resistance (Fig. 9F; Table 1). The expression of E- and N-cadherin in HK-2 cells transfected with each MT-3 mutant construct was assessed (Fig. 10). While the expression the constructs that produced domes in culture expressed high levels of E-cadherin, constructs containing only the N-terminal domain of MT-3 were also able to highly express this cadherin, despite the lack of the ability to form domes. The repression of N-cadherin, however, required the presence of the C-terminal domain (Fig. 10). The expression of connexin 32 required the presence of both domains with each domain being able to support intermediate levels of connexin 32 (Fig. 11).

**Table 1:** Transepithelial resistance and dome formation in HK-2 cells overexpressing MT-3 with altered protein domains.

Construct	MT Protein per μg protein	Domes per Field	Trans Epithelial Resistance (Ohm-cm^2^)
MT-3-NT Mutant			
#1	3.2 ± 0.25	6.2 ± 0.8	426 ± 36
#2	4.6 ± 0.31	3.5 ± 1.2	493 ± 42
#3	2.8 ± 0.29	3.3 ± 1.0	506 ± 42
MT-3ΔCT			
#1	3.7 ± 0.40	0	17 ± 12
#2	3.8 ± 0.11	0	15 ± 11
#3	2.2 ± 0.16	0	18 ± 18
MT-1E-NT			
#1	5.1 ± 0.72	0	22 ± 17
#2	3.6 ± 0.45	0	23 ±18
#3	4.2 ± 0.33	0	18 ± 9
MT-1E-CT			
#1	3.3 ± 0.26	9.8 ± 0.7	623 ± 31
#2	1.9 ± 0.08	6.7 ± 0.6	446 ± 33
#3	3.3 ± 0.33	4.4 ± 0.2	594 ± 23

### Effect of Forced E-cadherin Expression on HK-2 Vectorial Active Transport, N-Cadherin Expression, and Cell Morphology

**Fig 12:**
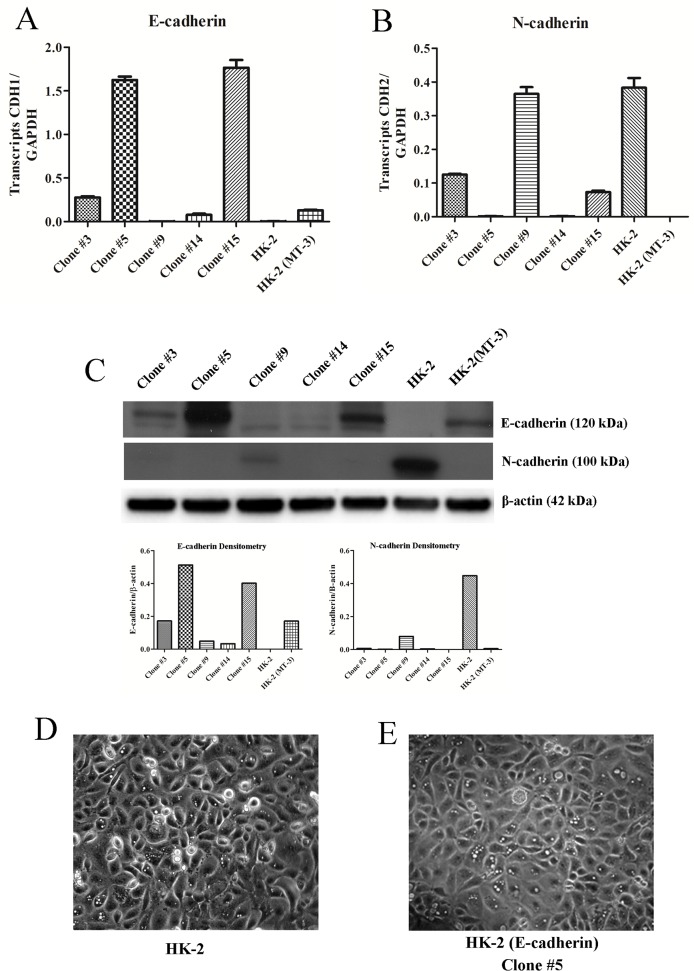
The effect of forced overexpression of E-cadherin on the expression of N-cadherin and on dome formation in HK-2 cells. E-cadherin was stably transfected into the HK-2 human proximal tubule cell line and individual clones were isolated and assessed for E-cadherin and N-cadherin expression and the formation of domes. A) Expression of E-cadherin mRNA in five individual clones, HK-2 cells and the MT-3 stably transfected HK-2 line, HK-2 (MT-3) assessed quantitatively with real-time PCR and normalized to the levels of transcripts of glyceraldehyde phosphate dehydrogenase; B) Expression of N-cadherin mRNA; C) Expression of E- and N-cadherin protein in each isolated clone, D) and E) Morphology of the highest expressing E-cadherin clone (E) in comparison to the parental HK-2 cells (D) showing the absence of dome formation.

It was previously shown that the stable transfection of HK-2 cells with MT-3 resulted in the re-establishment of vectorial active transport with a corresponding induction of E-cadherin and repression of N-cadherin gene expression [31]. In this study, E-cadherin was transfected into the HK-2 cells to determine if the expression of E-cadherin, in the absence of MT-3 expression, could restore vectorial active transport. It was also determined if E-cadherin expression would alter cell morphology or the expression of N-cadherin in the HK-2 cells. Five independent clones were selected and characterized for their expression of E-caherin mRNA and protein. Two clones (5 and 15) displayed an elevated expression of E-cadherin, two a very modest elevation (3 and 14) and one (9) no detectable alteration in expression of E-cadherin mRNA and protein (Fig. 12A,C). An analysis of N-cadherin mRNA expression showed that N-cadherin expression was highly repressed in the two isolates of HK-2 cells that expressed an elevated level of E-cadherin expression (Fig. 12A,B). N-cadherin mRNA was modestly repressed in the two isolates of HK-2 cells that expressed very modest elevation of E-cadherin expression (Fig. 12A,B). There was no alteration in the level of N-cadherin mRNA from that found in HK-2 cells for the transfected HK-2 cell clone (#9) that failed to increase their level of E-cadherin (Fig. 12A,B). The correlation between the expression of E-cadherin mRNA and the repression of N-cadherin mRNA was not as pronounced for the corresponding proteins (Fig. 12C). In agreement with the mRNA expression data, it was shown that whenever E-cadherin protein was expressed, there was no expression of the N-cadherin protein.

The results also demonstrated that transfection of the HK-2 cells with E-cadherin did not result in the re-establishment of vectorial active transport as evaluated by both dome formation or the development of a transepithelial resistance across the monolayer. None of the 5 independent clones showed any evidence of dome formation by the cell monolayers or developed a transpithelial resistance above a blank filter control (data not shown). The morphology of the cells was also not altered by transfection of E-cadherin into the HK-2 cell lines an noted by a similar morphology between wild type HK-2 cells and clone 5 which had the highest level of E-cadherin protein expression (Fig. 12D,E). This result also correlates with constructs containing the N-terminal but lacking the C-terminal domain being able to support E-cadherin expression but not sufficient enough for vectorial active transport (Fig. 10).

## Discussion

EMT is a developmental process where reorganization of the actin cytoskeleton, a loss of apical-basolateral polarity, and loss of cell-to-cell contact, results in conversion of an epithelial cell to a mesenchymal cell. The repression of the epithelial marker, E-cadherin, and upregulation of the mesenchymal marker, N-cadherin, are gene expression changes typically observed during EMT [39]. As detailed in the introduction, the HK-2 cell line has seen frequent use as a model to study the process of EMT in the human kidney. This laboratory previously reported that the HK-2 cell line had a very low expression of E-cadherin compared to N-cadherin, an absence of tight junction mediated paracellular barrier function, and a failure to maintain vectorial active transport [30, 31]. These would be features that suggest that the HK-2 cell line may have undergone an appreciable level of EMT during the isolation of the cell line. However, these studies used one of a variety of growth media employed for growth of the HK-2 cell line and therefore may not have been representative of other reports in the literature that use this cell line. Thus, the first goal of the present study was to confirm the levels of expression of the E- and N-cadherin, tight junctional integrity, and vectorial active transport in the HK-2 cell line as a function of growth media composition. The results of this study confirmed that the HK-2 cells expressed a very low amount of E-cadherin mRNA and protein and that this expression was largely independent of the composition of the growth media. Similarly, the expression of N-cadherin was very high compared to that of E-cadherin. The HK-2 cell monolayer exhibited no transepithelail resistance on any of the growth media tested, confirming a lack of tight junctional integrity. It was also demonstrated that the HK-2 cell line did not form “domes”, a feature of vectorial active transport, on any of the tested growth mediums. The formation of domes is a hallmark of cultured renal epithelial cells that retain the *in situ* property of vectorial active transport [34]. These out-of-focus areas of the cell monolayer, seen upon light microscopic examination, represent raised areas where fluid has become trapped underneath the monolayer owing to active transport of ions and water across the cell monolayer in an apical to basolateral direction. These results show that the HK-2 cell line, regardless of the conditions used for growth, have a low expression of E- compared to N-cadherin, an absence of tight junctions, and do not form “domes”.

There is agreement in the literature that the renal proximal tubule does express N-cadherin in line with a developmental derivation from mesenchyme [40–43]. However, the literature is somewhat less clear regarding the expression of E-cadherin. In the developing kidney, it has been shown that the early progenitors of the proximal tubules express cadherin 6 and not E-cadherin [44]. Then, as the proximal tubules mature, cadherin-6 (also called K-cadherin) is down-regulated and E-cadherin is then detected in the mature proximal tubules of the adult kidney [44]. In this regard, cadherin-6 can be looked upon as a marker for the fetal proximal tubule. Despite this convincing study, there is some discrepancy in the literature regarding the expression of E-cadherin in the proximal tubule, with some reports indicating no expression [41,42, 45–47], others low expression well below that of N-cadherin [40, 48, 49–51], and some with expression without indication of level [52], [53]. Due to this discrepancy in E-cadherin expression, the present study examined the expression of E- and N-cadherin in the human kidney proximal tubule. It was shown using immunohistochemistry on three independent specimens of human kidney that the proximal tubules had expression of both E- and N-cadherin protein. The study was also extended to the level of mRNA expression by microdissection of proximal tubules from 3 independent specimens and determining the expression of E- and N-cadherin by real time PCR. It was found that there was expression of both E- and N-cadherin mRNA in the proximal tubules. The proximal tubules chosen for microdissection were those in close proximity to glomeruli, greatly minimizing any chance for contamination with other types of tubular elements. A plausible reason for the discrepancy in the literature regarding the expression of E-cadherin in the proximal tubule is that most studies relied on immunohistochemistry, both peroxidase and fluorescent based, in attempts to detect and compare the intensity of staining between antibodies for E- and N-cadherin. While it is possible to compare the intensity of staining within each antibody across the differing elements of the kidney, it is not possible to compare the intensity of staining generated between the E- and N-cadherin antibodies and draw conclusions regarding levels of expression. Each antibody will have its own characteristics of antigen binding and staining can also vary further between antibodies as a function of tissue fixation, antigen retrieval techniques, and incubation conditions to name but a few variables. It is extremely difficult, if not impossible, to compare the level of expression of E-cadherin to N-cadherin using immunohistochemical analysis. Thus, the present study shows that both E- and N-cadherin mRNA and protein are expressed in the human renal proximal tubule.

The present results suggest that the HK-2 cell line has already undergone an appreciable degree of EMT. This is based on several observations. First, the HK-2 cell line does not form “domes” in cell culture. This is in contrast to primary cultures of human renal epithelial cells, isolated and cultured by several different laboratories that have been shown to form “domes” [54, 55–56, 32]. In fact, the primary culture used in the isolation of the HK-2 cell line was stated to form “domes” prior to being used in the transfection and immortalization procedure [14]. Dome formation in epithelial cell cultures is acceptable presumptive evidence of the following processes that are required for its expression: functional plasma membrane polarization, formation of occluding junctions (tight junctions), and vectorial transepithelial active ion transport [57]. Studies from this laboratory demonstrated that the HK-2 cell line does not generate a transepithelial electrical resistance or possess tight junctional sealing strands, both indicative of a loss of tight junctions between adjacent cells [34]. Second, the present study confirms that the HK-2 cell line has undergone an E-cadherin to N-cadherin shift when compared to the HPT cells. The present study quantified the difference in E-cadherin and N-cadherin between the HPT and HK-2 cells. This showed high levels of E-cadherin in the HPT cells and very low levels in the HK-2 cell line. The reverse was shown for N-cadherin expression, but the magnitude of the difference was not as large. In addition, when compared to the HPT cells, the HK-2 cell line has increased expression of cadherin-6 (K-cadherin), a marker associated with the proximal tubule of the developing kidney [44, 31]. This finding suggests a more mesenchymal differentiation of the HK-2 cells based on the known development of the proximal tubule [44]. Third, the human proximal tubule is known to possess gap junctions between adjacent cells. The present study demonstrated that the HPT cells expressed connexin 32; whereas, the HK-2 cell line had a much reduced expression of this connexin. Thus, the present results suggest that the HK-2 cells already displays those features of a cell having undergone appreciable EMT.

However, the results should not be interpreted to indicate that the HK-2 cell line is an unacceptable model for the study of the overall process of EMT. Too often EMT is looked upon as an all-or-none process rather than a graded series of events. The HPT cell line and all other primary mortal cultures of cells retaining proximal tubule character have lost their brush border. This could be looked upon as a very early feature of EMT that occurs upon placing the cells into culture. Similarly, the HK-2 cell line could be looked upon as a model for a proximal tubule cell that has lost early features associated with EMT such as cell-to-cell contact, a loss of apical to basolateral polarity and cadherin switching. However, the HK-2 cells retain many other differentiated features of the proximal tubule. As detailed in the introduction, these include: proteins such as alkaline phosphatase, gamma glutamyltranspeptidase, leucine aminopeptidase, acid phosphatase, cytokeratin, α_3_β_1_ integrin and fibronectin; and functional markers such as, the cAMP responsiveness to parathyroid hormone and not antidiuretic hormone, Na^+^ dependent, phlorizin sensitive glucose transport, and the ability to accumulate glycogen. As such, the HK-2 cell line could be looked upon as a model system where EMT has progressed with regard to a loss of cell junctions and the E- to N-cadherin switch, but with the retainment of many features of proximal tubule differentiation. The HK-2 cell line may be very valuable in elucidating pro-fibrotic factors and in defining the changes necessary for the HK-2 cells to gain further mesenchymal differentiation.

There is also convincing evidence that the differences between the HPT and HK-2 cell line is not an anomaly of the isolation protocol used to develop the HK-2 cell line. The HK-2 cell line is derived from a single clone of cells following transfection with the HPV E6/E7 genes as designated in the ATCC product sheet and the original publication [14]. Thus, a simple explanation for the finding that HK-2 cells have no tight junctions and have undergone an E- to N-cadherin switch is that the HK-2 cell line was derived from an aberrant single cell that possessed these properties in the original primary culture used for isolation of the cell line. Under this scenario, the originating cell would not have been representative of the cells within the primary culture that were able to form domes. However, if the above scenario were the case, the HK-2 cell would not be expected to be able to undergo MET and regain the original characteristics of the cells of the primary culture that are able to form domes. Studies from this laboratory have shown that the HK-2 cells can undergo MET and regain the features expected of primary cultures of human proximal tubule cells [30, 31]. This was accomplished by the stable transfection of the MT-3 gene into the HK-2 cell line. The HK-2 cells transfected with the MT-3 gene regained the ability to form domes in culture, displayed increased transepithelial resistance, and a switch from N-cadherin to E-cadherin expression. The present studies also demonstrated that the MT-3 transfected cells regained the expression of the connexin 32 gap junction protein, similar to that found in primary cultures. These studies show that the HK-2 cell line can undergo MET and convert back to those phenotypic and genotypic properties of the original primary culture. In addition, the ability of the MT-3 gene to induce MET in HK-2 cells provides a model for the study of MET in the proximal tubule and also might uncover mechanistic approaches to halt the pro-fibrotic microenvironment of the kidney if it indeed interstitial fibrosis, wholly or partially, develops from EMT of tubular epithelium. There appears to be many more models for the study of renal EMT than that of MET in the adult kidney.

The finding that MT-3 elicited MET-like changes in HK-2 cells was not based on a mechanistic hypothesis regarding EMT or MET. Rather, the laboratory was studying the possible role of the MT-3 gene in cadmium-induced renal toxicity. There is no other existing literature to suggest a role of MT-3 in renal EMT or MET. There is also no literature on how MT-3 might interact with the HPV E6 and E7 genes to promote MET in HK-2 cells. However, the MT-3 gene and protein has two unique sequences that help define the epitope of the MT-3 gene and protein that participates in EMT and MET. The MT-3 isoform is unique among the MT gene family and these differences with other family members have been highlighted in past reports from this laboratory [30, 31, 58, 59]. Of importance in the present study is that MT-3 possesses 7 additional amino acids that are not present in any other member of the MT gene family, a 6 amino acid C-terminal sequence and a Thr in the N-terminal region [60–62]. The current study shows that the unique C-terminal amino acid sequence of MT-3 was required to re-establish vectorial active transport and the shift in E- and N-cadherin expression for the HK-2 cells. This would be the first study to define a functional significance to this unique sequence of the MT-3 protein. The only other study was one designed to define how the C-terminal insert in MT-3 would alter metal binding characteristics compared to other members of the MT gene family [63]. This study demonstrated that the C-terminal hexapeptide insert would render the MT-3 α-domain in a looser conformation and lowers the stability of the metal-thiolate cluster; an alteration that would render the metal binding site more accessible for exchange mechanisms with potential protein partners. Overall, the present study demonstrates the essential nature of the C-terminal sequence by both deletion of the sequence from the MT-3 gene and by insertion of the sequence into the MT-1E isoform, which resulted in the loss of dome formation and the establishment of dome formation in transfected HK-2 cells, respectively.

## Supporting Information

S1 Fig Effect of growth surface on E- and N-cadherin expression in HPT and HK-2 cells. E-cadherin (A) and N-cadherin (B) protein expression was assessed following growth on either tissue culture treated dishes (TCTD) or 30 mm Transwell inserts (Corning, Tewksbury, MA). B-actin was used as a loading control and for densitometric normalization. Densitometry is shown below the corresponding blot.
(TIF)
