# Supplementary figures and images for: Cadherin Expression, Vectorial Active Transport, and Metallothionein Isoform 3 Mediated EMT/MET Responses in Cultured Primary and Immortalized Human Proximal Tubule Cells
Source: PLoS One. 2015 Mar 24;10(3):e0120132. doi: 10.1371/journal.pone.0120132 (PMC4372585; doi:10.1371/journal.pone.0120132)

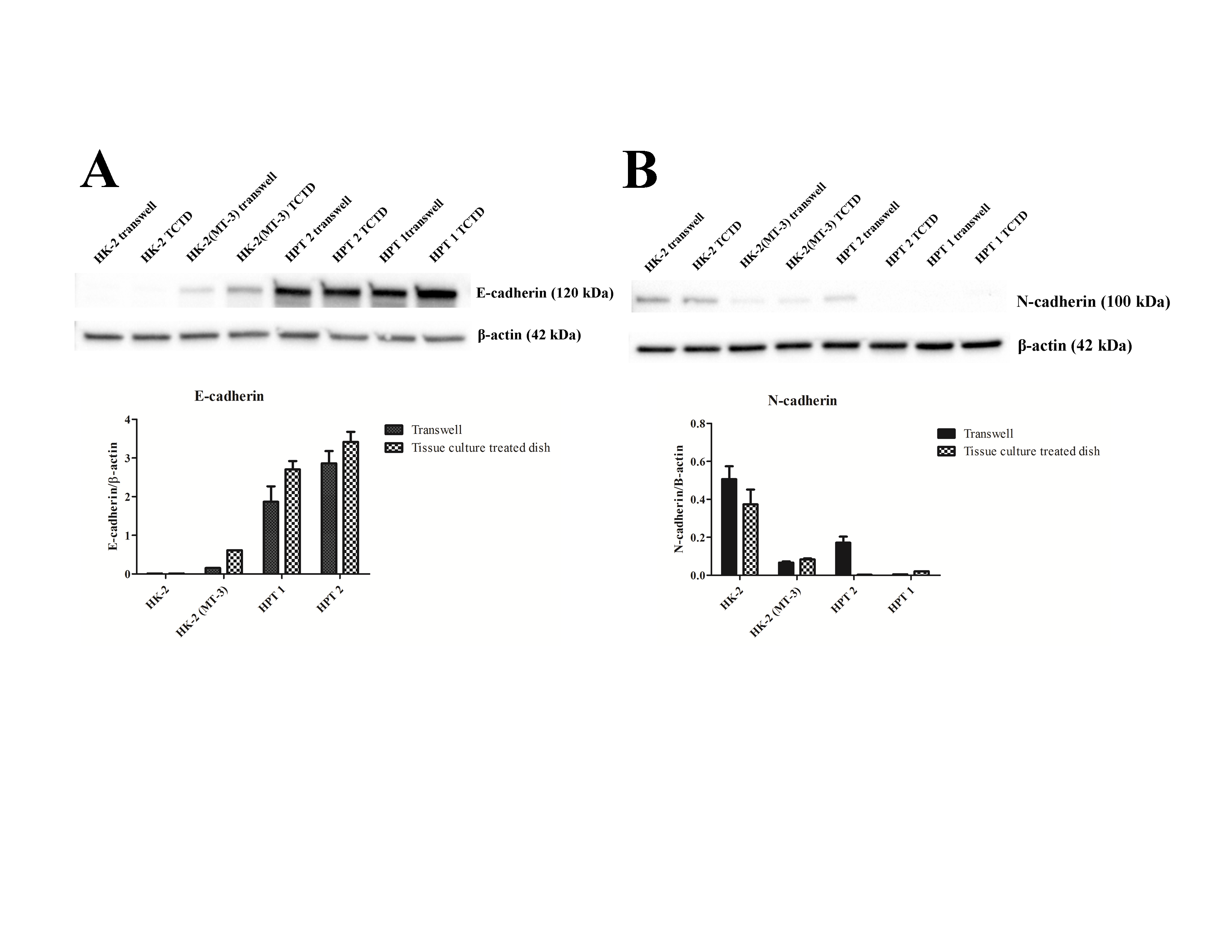

Supplement: S1 Fig — E-cadherin (A) and N-cadherin (B) protein expression was assessed following growth on either tissue culture treated dishes (TCTD) or 30 mm Transwell inserts (Corning, Tewksbury, MA). B-actin was used as a loading control and for densitometric normalization. Densitometry is shown below the corresponding blot. (TIF) [file pone.0120132.s001.tif]
